# Numerical Analysis of Microcrack Propagation Characteristics and Influencing Factors of Serrated Structural Plane

**DOI:** 10.3390/ma15155287

**Published:** 2022-07-31

**Authors:** Xing Zhang, Hang Lin, Jianxin Qin, Rihong Cao, Shaowei Ma, Huihua Hu

**Affiliations:** 1 School of Resources and Safety Engineering, Central South University, Changsha 410083, China; zhangxing1994@csu.edu.cn (X.Z.); mrqinjianxin@163.com (J.Q.); 18229997417@163.com (R.C.); 2 Hunan Provincial Communications Planning, Survey and Design Institute, Changsha 410200, China; huhuihuahnjg@126.com

**Keywords:** numerical calculation, serrated structural plane, shear properties, crack evolution

## Abstract

The serrated structural plane is the basic unit of structural plane morphology. However, the understanding of its internal stress distribution, failure mode and crack evolution law was not clear enough in previous studies. In this paper, the shear mechanical properties of the serrated structural planes were studied by numerical simulation, and the crack evolution law of the serrated structural planes and the effects of four microscopic parameters on the shear properties were analyzed. The results show that: (1) the number of microcracks increases with the increase in normal stress; the crack expansion rate is slow before the shear stress reaches the peak. After the shear stress reaches the peak, the crack expansion rate continues to increase, and the microcracks keep sprouting and expanding, and the number of microcracks tends to stabilize when the shear stress reaches the residual shear strength. (2) The particle contact stiffness ratio $k_{n}{}^{*}/k_{s}{}^{*}$ and parallel bond stiffness ratio $k_{n}/k_{s}$ were negatively correlated with the shear strength; and the particle contact modulus E and parallel bond modulus $E^{*}$ were positively correlated with the shear strength. As the particle contact modulus E and parallel bond modulus $E^{*}$ increase, the peak shear displacement gradually decreases. The parallel bond stiffness ratio $k_{n}/k_{s}$ has a negative correlation with the peak shear displacement. This study is expected to provide theoretical guidance for the microscopic parameter calibration and shear mechanical analysis of serrated structural planes. (3) Several XGBoost, WOA-XGBoost, and PSO-XGBoost algorithms are introduced to construct the quantitative prediction model, and the comparative analysis found that WOA-XGBoost has the best fitting effect and can be used for the prediction of shear strength. When using this model to calculate the weight shares of micro-parameters, it was found that $k_{n}{}^{*}/k_{s}{}^{*}$ has the greatest influence on shear strength, followed by $E^{*}$; E and $k_{n}/k_{s}$ had the least influence.

## 1. Introduction

**Figure 1:**
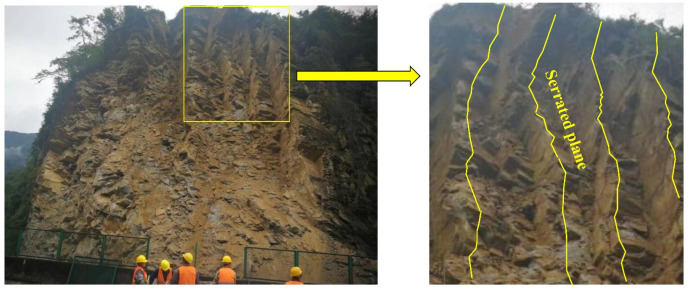
The distribution of one serrated structural plane of a slope.

Rock discontinuity is an engineering medium widely existing in nature, and its stability is controlled by its structural plane [1,2,3]. As a result, the engineering construction and its stability have an indivisible relationship with the strength of the structural plane and deformation characteristics [4,5,6]. The studies on shear mechanical behavior on the structural plane and damage rules have important theoretical value and significant practical engineering utility [7,8,9]. According to the fluctuation degree of structural plane, the structural plane can be classified one of into four categories: straight, stepped, serrated, or wavy [10,11]. Among them, the serrated structural plane is the basic unit of structural plane patterns, which can form new ones with other structural plane patterns by combining different serrated structural planes [12,13]. The serrated structural plane is generally formed by the mutual connection of the rock bridge between two groups of cracks. Serrated structure planes in natural rock mass are shown in Figure 1, where the joints have an obvious serrated shape.

Although studies on the shear properties of structural plane have been carried out for years, the macroscopic mechanical changes during the shearing of the structural plane can only be obtained under the limitations of experimental conditions [14,15,16,17]. Due to the rapid development of computer technology, it has been a trend to apply numerical simulation technology to the study of rock discontinuity mechanics [18,19,20]. Some scholars determined the shear behaviors of structural planes by using numerical simulations. Based on the characteristics of discontinuity, heterogeneity and anisotropy of rock structure [21,22,23], it is most suitable to choose particle follow code method (PFC) to conduct numerical simulations [24,25]. PFC can conduct research on the mechanical characteristics of a rock structure from the micro level and simulate the mechanical behavior of rock in different working situations, which can provide a supplement to and comparison with laboratory testing [26,27]. Bahaaddini, Sharrock and Hebblewhite [28] adopted the value of discrete elements PFC2D to simulate the shearing behavior of rock joints. Jiang, Liu, Crosta and Li [29] adopted the calculation method of discrete element value to study the influences on rock shearing behavior taken by geometry patterns of rock. Yang and Qiao [30] conducted numerical researching of discontinuous jointed granite material by using the joint specimen. At present, most numerical simulation studies on structural surfaces are mainly aimed at the macroscopic mechanical behavior in the shearing process, such as shear stress–strain curves, stress contour distribution, displacement contours, etc. [31,32,33]. There are few studies on the appearance characteristics, failure forms and crack propagation laws [34,35,36,37]. Therefore, this paper adopts the PFC numerical calculation method to simulate the shear mechanical behavior of the serrated structural plane, and discusses the response law of the corresponding influencing factors.

## 2. Establishment of the Numerical Calculation Model

**Figure 2:**
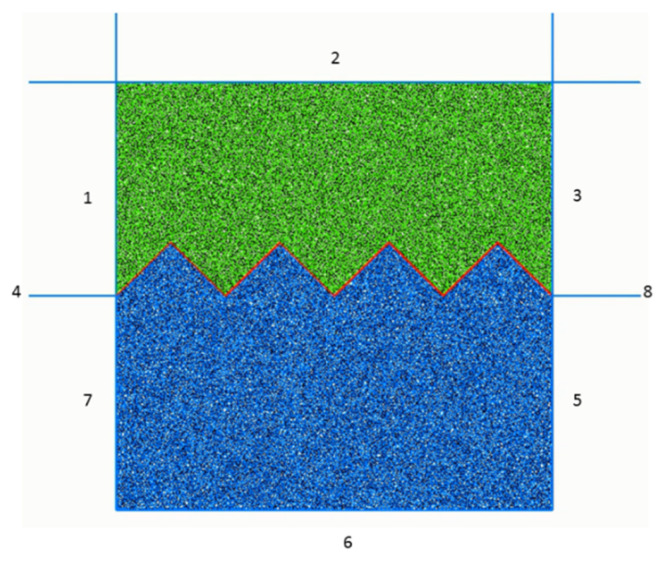
The numerical mode of PFC2D in a serrated structural plane (1–8 are the numbers of the walls respectively).

In this study, the PFC2D was used to establish a numerical model of the serrated structural plane, as shown as Figure 2, with the size of 100 × 100 mm. PFC simulates the motion by interacting with particles using the discrete element method, which is widely used to study the mechanical properties of rocks and rock-like materials [26,27].

The process of numerical model establishment can be summarized as follows: (1) Firstly, 8 walls were defined in PFC as the boundary of the shear box, with the upper walls (1#, 2#, 3#, 4#) as the “top” group and the lower walls (5#, 6#, 7#, 8#) as the “bot” group. In this model, the shear box is composed of two parts: walls 1, 2, and 3 represent the upper shear box; and walls 5, 6, and 7 represent the lower shear box. (2) Then, within the boundaries of shear box, 10,426 particles were randomly generated by FISH language, and the radii of particles in the model were 0.4–0.64 mm; the porosity was 0.1. The parallel bond model commonly used to simulate rocks was used as the contact model between particles. (3) Subsequently, the DFN fractures representing serrated joints were added to the generated initial model to generate the final numerical calculation model. (4) Finally, through repeated calibration trial calculations [38], the micro-parameters of the structural plane model were obtained.

The direct shear test simulation adopts servo loading and the displacement control method. The servo program was prepared by FISH language to keep the normal stress constant. Then, walls 1, 2, and 3, which constitute the upper shear box, were made to move horizontally at a constant shear displacement rate to simulate the direct shear mechanical behavior.

The test results under normal stress of 1.2 MPa in the literature [39] were taken as the calibration object, and the micro-parameters of the parallel bond model were repeatedly calibrated based on the trial-and-error method. Subsequently, the parameter values were obtained as shown in Table 1.

**Figure 3:**
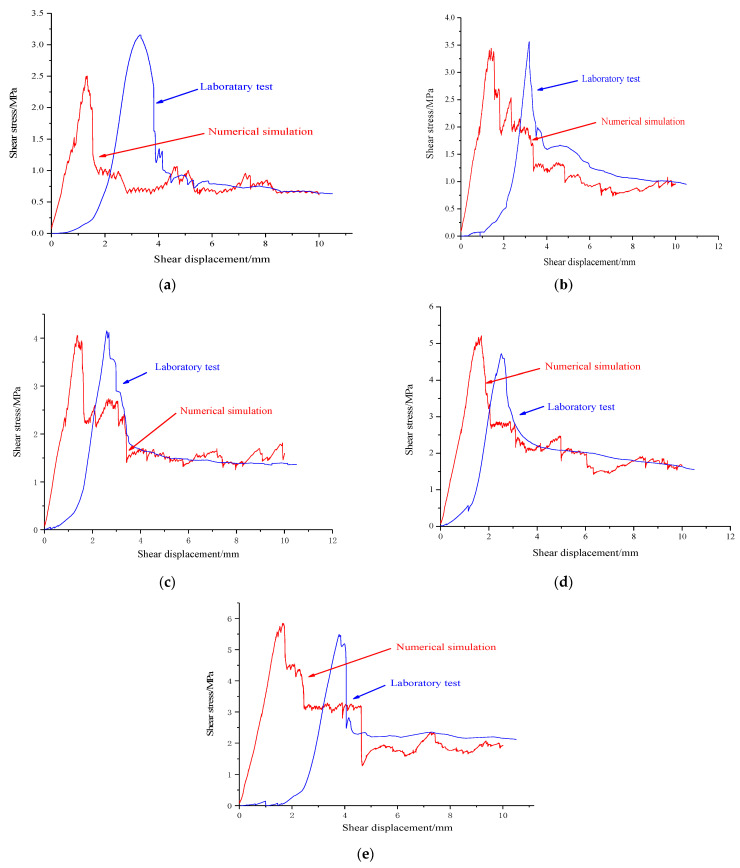
Numerical simulation results and laboratory testing results [39] with different normal stress of serrated structural plane. (**a**) Normal stress = 0.4 MPa. (**b**) Normal stress = 0.8 MPa. (**c**) Normal stress = 1.2 MPa. (**d**) Normal stress = 1.6 MPa. (**e**) Normal stress = 2.0 MPa.

Numerical simulation results and laboratory test results are shown in Figure 3. The numerical simulation curve of PFC2D is not only in good agreement with the test results under the normal stress of 1.2 MPa adopted for calibration, but also very close to the test results of other same samples under different normal stress conditions.

The shear mechanical parameters under test and simulation conditions were calculated by Mohr–Coulomb criterion, as shown in Table 2.

The cohesion and internal friction angles are also similar. Under the different normal stress, the shear stress–shear displacement curve of the numerical mode shows the obvious characteristics of shear hardening before the peak value and shear softening after the peak value. The main differences between numerical simulation results and testing results are in the initial loading stage, mainly because of there is no close pressing between the horizontal loading device and shearing boxes of direct shear apparatus in this stage. Therefore, it needs to make contact adjustments in the initial loading stage, and at this moment, it is reflected on the curve that with the increments in shear displacement, the increment in shear stress of the structural plane is small. With the increment in shear displacement, the serrated structural plane gets continuous contact coupling, and a certain degree of squeezing deformation happens in the structural plane. The structural plane starts to play the rule of shear–resistance performance, and the shear stress is increased rapidly with the incrementation of shear displacement. In the numerical model, the upper and lower shear boxes of structural planes are close in the initial loading stage. Therefore, the contact coupling stage does not exist in the initial stage.

## 3. Crack Evolution Rule in the Shear Process of the Structural Plane

### 3.1. The Crack Development Rule

**Figure 4:**
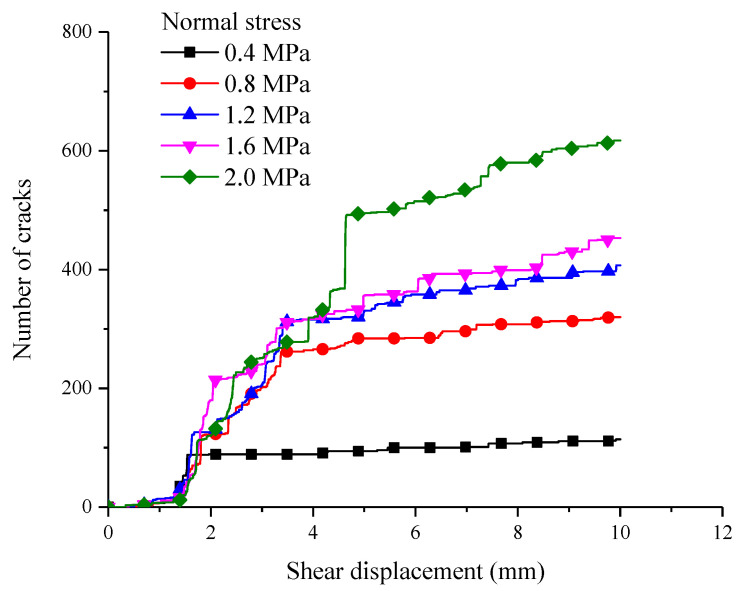
The relationship between the number of microcracks and shear displacement.

During the shear process, microcracks would be generated due to damage in the interior of rock, but in the laboratory testing, the microcrack expansion rule cannot be obtained. In PFC, the number, location and type of microcracks can be tracked by the built-in FISH program, as shown in Figure 4. In the initial loading stage, when the shear displacement is 1–1.5 mm, there is almost no microcrack generated. when the shear displacement is more than 1.5 mm, the number of microcracks is increased linearly along with the increase in shear displacement. When the number of microcracks reaches a certain value, the number of microcracks is not increased any more while there is a consistent increment in shear displacement.

By comparing and analyzing the Figure 3 and Figure 4, we can see that the increment rate of cracks is slow before shear stress reaches its peak. Once the shear stress reaches its peak value, the number of microcracks increases rapidly till the structural plane comes to the residual strength stage. Subsequently, due to the slip damage happening to the structural plane, the number of microcracks stops increasing. Taking the normal stress of 0.4 MPa, for instance, when the shear displacement is 2.0 mm, the shear strength gets into the residual stage, and the number of microcracks is about 100. When the shear displacement is between 0.8 and 2.0 MPa and the shear displacement is about 5 mm, the shear strength gets to the residual stage, and the number of microcracks in the interior shear box is basically stable, which are about 280, 320, 350, 400, and 500, respectively. During the shearing process, the bigger the normal stress, the higher the number of microcracks. This is because the bigger the normal stress, the easier the serrated on the structural plane is to be damaged.

### 3.2. The Evolution of Microcracks and Contact Force of Structure Plane

**Figure 5:**
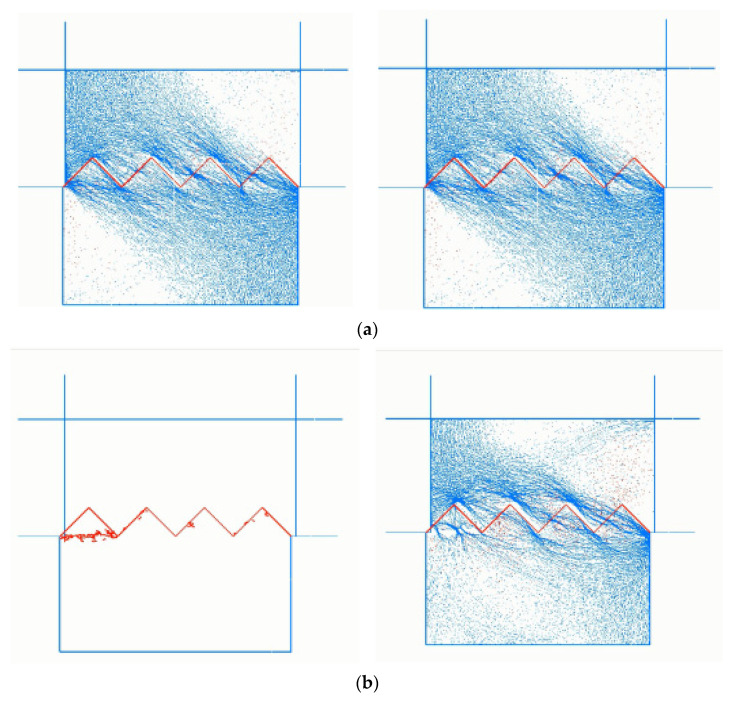

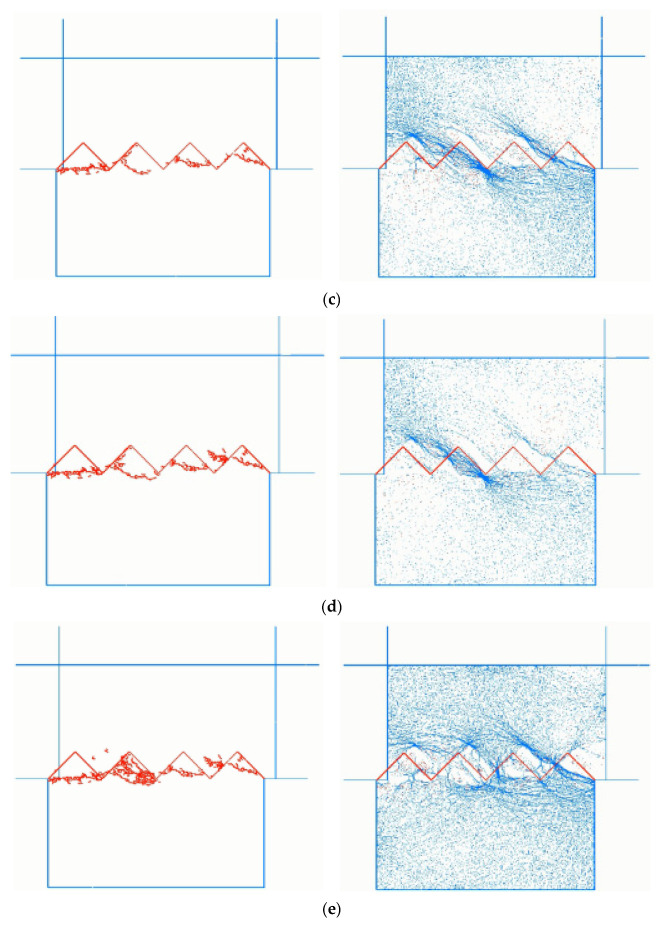

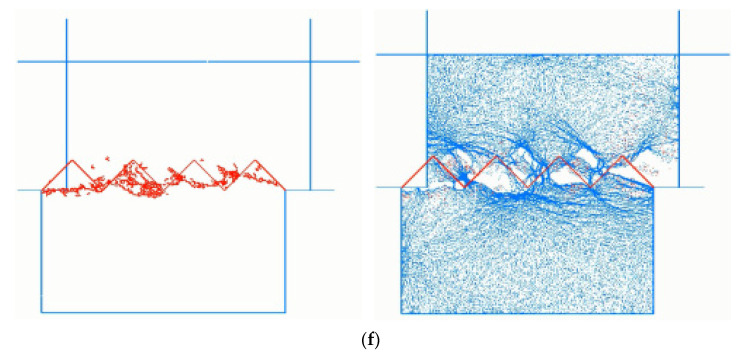
Distribution of cracks expansion and contact force with different shearing displacement. (**a**) Shear displacement = 1 mm. (**b**) Shear displacement = 2 mm. (**c**) Shear displacement = 3 mm. (**d**) Shear displacement = 4 mm. (**e**) Shear displacement = 5 mm. (**f**) Shear displacement = 10 mm.

The damage characteristics of the serrated structure plane are reflected by the distribution situations and number of microcracks, and the contact force between particles through dynamic supervising. The recorded distribution situations of cracks, expansion and contact force of structure planes during the shearing process are shown in Figure 5.

It can be seen in Figure 5 that at the beginning of shearing, the normal stress is greater than the shear stress, and the force chain presents a near-normal propagation direction. As the shear displacement increases, the shear stress also increases, and the shear load is transmitted between particles through the force chain. At this time, the value of shear stress increases, and the normal stress remains unchanged. Therefore, the direction of the combined force of the two forces gradually changes to the near horizontal direction. During the shear process, because the lower shear box is constrained, and the upper shear box is pushed and cut, the propagation direction of the force chain is shown as the shear direction of the upper test block, and the propagation direction of the force chain in the lower test block is the inverse shear direction. With the increase in shear displacement, the distribution of contact force on the serrated surface is consistent with the growth of microcracks on the structure surface. At the beginning of shearing, the contact force chain mainly concentrates on the serration root and becomes more and more concentrated with the increase in shear displacement. It should be noted that the force chain initially concentrated on the left-most serrated root. As the shear process went on, the force on the serration root became larger and larger, which eventually resulted in a large number of cracks at the root of the serration. After the cracks were connected, the macroscopic damage of the serration was shown, that is, the serration was gnawed. After the first saw tooth was destroyed, the force chain began to focus on the root of the second saw tooth. Later serrations were gnawed on the same principle. Finally, as the shearing behavior continued, the gnawed serration “large particles” were further ground up, and the grinding process of the “large particles” still provided some shear stress, so that the force chain occurred at these sites.

**Figure 6:**
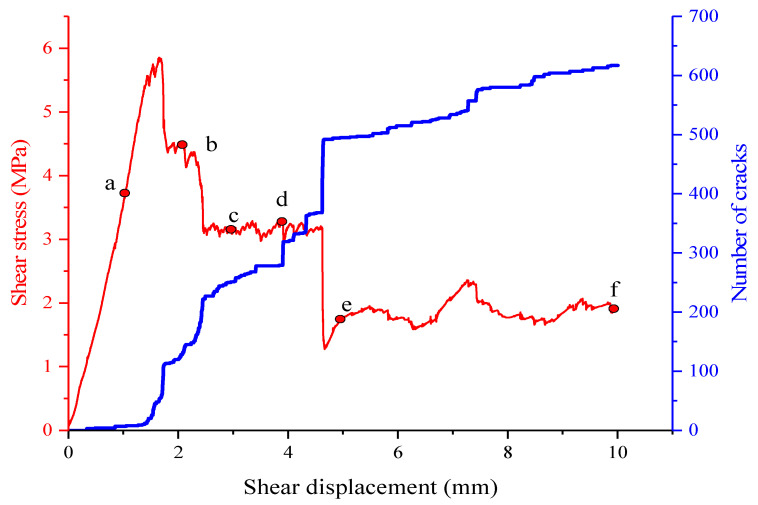
The shear stress and number of cracks in the serrated structural plane. (a, b, c, d, e, and f correspond to the monitoring points where the shear displacements are 1 mm, 2 mm, 3 mm, 4 mm, 5 mm and 10 mm, respectively).

The relationship between crack expansion and shear stress is recorded in Figure 6.

As can be seen from the figure, when the shear displacement is 1 mm, only a few of the microcracks in the root of serration appear in the structure plane. When the shear displacement is 2 mm at point b, the shear stress reaches its peak value. At this time, the number of microcracks is increased little bit more than in the point a, which is about 300, and most are focused on the first root of the serration on the left, and they are related to each other. This shows that the first root of serration has been cut completely, which is also the main reason for shear stress reduction from the peak level. Along with the shear displacement being increased to 3 mm, the number of microcracks is increased to about 270, and the cracks have been expanded at the second, third and fourth roots of serration. All cracks in the root tend to be connected, and the shear stress is reduced further. When the shear displacement is 4 mm, the shear stress is almost unchanged, and the number of microcracks is increased to be about 320. The cracks in all roots of serration are developed further but are not completely connected. When the shear displacement is 5 mm, the increasing speed of cracks number is high, and it reaches about 500. Now, the cracks in all roots have been totally connected and the shear stress is almost stable, reaching residual strength. The serration cut off is ground in further, and the number of microcracks is increased in further as well.

## 4. Effect of Micro-Parameters on Shear Mechanical Behavior

### 4.1. Parallel Bond Stiffness Ratio $k_{n}/k_{s}$

**Figure 7:**
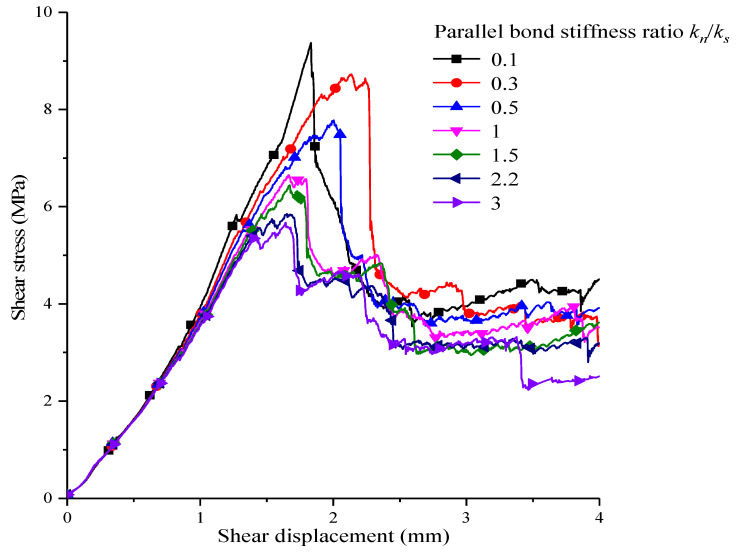
Shear stress–shear displacement curves of corresponding different parallel bond stiffness ratios.

The ratio of normal stiffness $k_{n}$ to shear stiffness $k_{s}$ in particle bonding is called the parallel bonding stiffness ratio, $k_{n}/k_{s}$. To discuss the influences on shear strength taken by the parallel bonding stiffness ratio, the $k_{n}/k_{s}$ value is changed, and meanwhile, the other micro-parameters are kept constant. By conducting direct shear simulation testing on serrated structural plane in the 2.0 MPa direct stress, the shear stress–shear displacement curve of corresponding different bonding stiffness ratio is obtained, which is shown as Figure 7. The parallel bonding stiffness ratio has a greater influence on the shear stress–shear displacement curve of the serrated structural plane. There is a negative correlation between the peak shear strength and the increase in the parallel bonding stiffness ratio. In the process of the parallel bond stiffness ratio being reduced to 0.1 from 3.0, the rate of increasing of the shear strength of the structural plane increases. After the parallel bond stiffness ratio becomes less than 1, the incrementation in shear strength is more obvious. Meanwhile, along with the reduction in parallel bond stiffness ratio, the slope of the shear stress–shear displacement curve is increased generally before reaching its peak. The shear displacement of shear strength becomes lower when reaching the peak, and the “drop” rate of the shear stress–shear displacement curve after reaching peak strength is faster, especially when the parallel bond stiffness is 0.1.

### 4.2. Particle Contact Stiffness Ratio $k_{n}{}^{*}/k_{s}{}^{*}$

**Figure 8:**
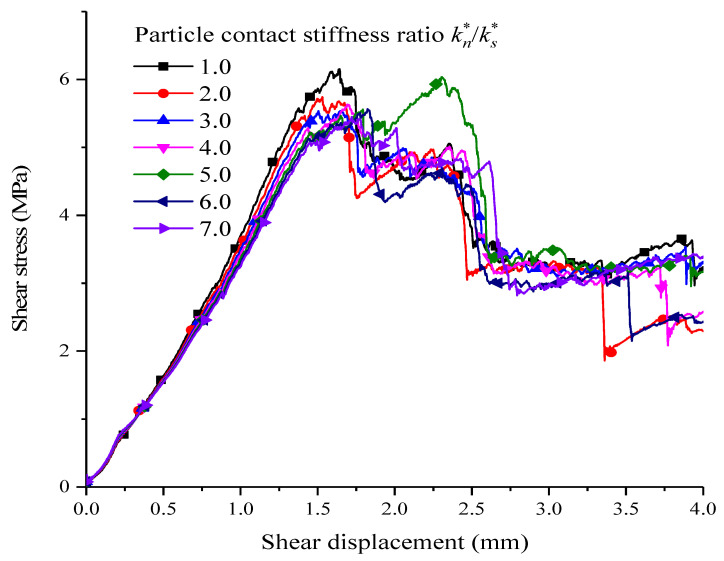
The corresponding shear stress–shear displacement relationships of different particle contact stiffness ratios.

The contact stiffness ratio $k_{n}{}^{*}/k_{s}{}^{*}$ between particles is the ratio of normal stiffness $k_{n}{}^{*}$ to tangential stiffness $k_{s}{}^{*}$ of the contact between particles. Through keeping the other parameters unchanged, the influences on shear stress and shear displacement taken by contact stiffness ratio $k_{n}{}^{*}/k_{s}{}^{*}$ between particles were simulated in groups of contact stiffness ratios, which is shown as Figure 8.

Although there are different shear stress and shear displacement curves for different contact stiffness ratios, the difference is not great, and the curve patterns are almost consistent; meanwhile, the shear strength of the corresponding peaks are almost the same. With the above stiffness ratios, the shear resistance strength reaches its maximum when the stiffness ratio is one, which is 6.2 MPa. The contact stiffness ratio has a certain influence on the slope of shear stress–shear strain before reaching its peak. When the contact stiffness ratio is less than three, the slope of the curve decreases gradually with the increase in $k_{n}{}^{*}/k_{s}{}^{*}$; that is, the elastic modulus decreases. The above analysis states the influences of contact stiffness strength between particles on shear resistance strength and elastic modulus are weak.

### 4.3. Particle Contact Modulus E

**Figure 9:**
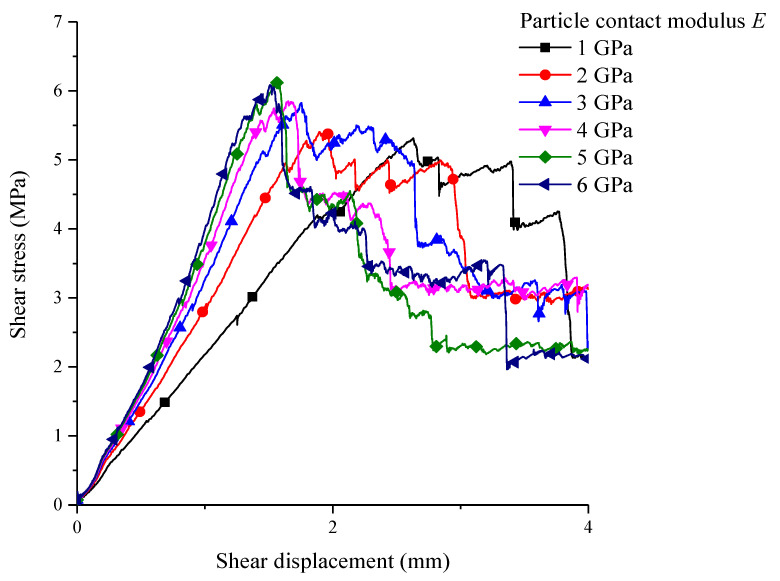
The shear stress–shear displacement curve under different values of particle contact modulus.

The contact modulus of particles is the elastic modulus. The corresponding shear stress–shear displacement curves of groups of contact modulus were obtained by only changing the contact modulus between particles, as shown as Figure 9.

The contact modulus between particles has a great influence on the shear characteristics of the structural plane, which is mainly reflected in the elastic modulus. As the contact modulus is increased to 6 GPa from 1 GPa, the slope of the corresponding shear stress–shear displacement curve increases generally, before reaching its peak. However, as the ratio becomes smaller, the corresponding slopes of the curves when the contact modulus is 5 or 6 GPa are almost the same. Meanwhile, the changes in contact modulus between particles have a certain influence on the shear resistance strength of the pattern. When the particles’ contact modulus is increased, the shear strength is increased as well. The range of increments is not big. When the contact modulus is 6 GPa for particles, the corresponding shear resistance peak is maximal: about 6.3 MPa.

### 4.4. Parallel Bond Modulus $E^{*}$

**Figure 10:**
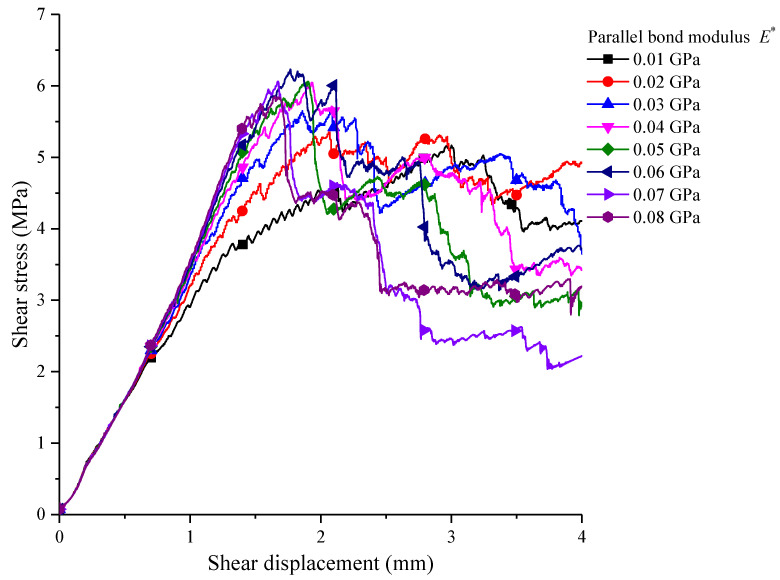
The corresponding shear stress–shear displacement curves with different parallel bond moduli.

The elastic modulus is $E^{*}$ between particles that are bonded by parallel bonding, which is called the parallel bonding modulus. Under the guarantee of other micro-parameters being unchanged, groups of shear stress–shear displacement curves corresponding to parallel bonding elastic moduli were obtained by changing the parallel bonding elastic modulus, which is shown as Figure 10.

The parallel bonding modulus has a great influence on the shear stress–shear displacement curve, especially with the incrementation of the parallel bonding modulus: the corresponding shear displacement is reduced when shear stress reaches its peak. The slope of the curve becomes greater generally before the peak, and with the increments in parallel bonding modulus, the peak displacement gets closer to 1.7 mm. As shown in Figure 10, the parallel bonding modulus has a certain influence on shear stress. When the parallel bonding modulus is increased to 8 GPa from 1 GPa, the shear resistance strength is increased overall, especially when the parallel bonding modulus is higher than 4 GPa. When the parallel bonding modulus reaches 6 GPa, the maximum peak shear resistance strength is about 6.3 MPa. Subsequently, the shear resistance strength is reduced tightly.

### 4.5. Effects of Micro-Parameters on Shear Strength of the Structural Plane

**Figure 11:**
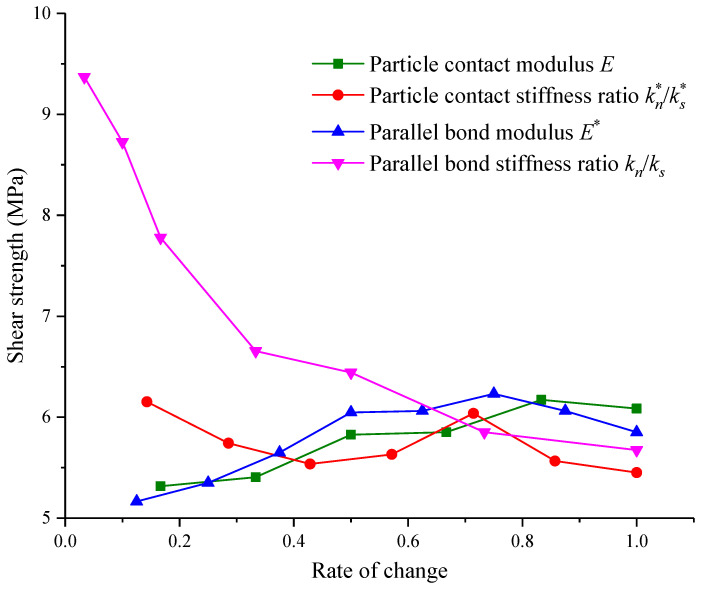
Effects of micro-parameters on shear strength.

**Figure 12:**
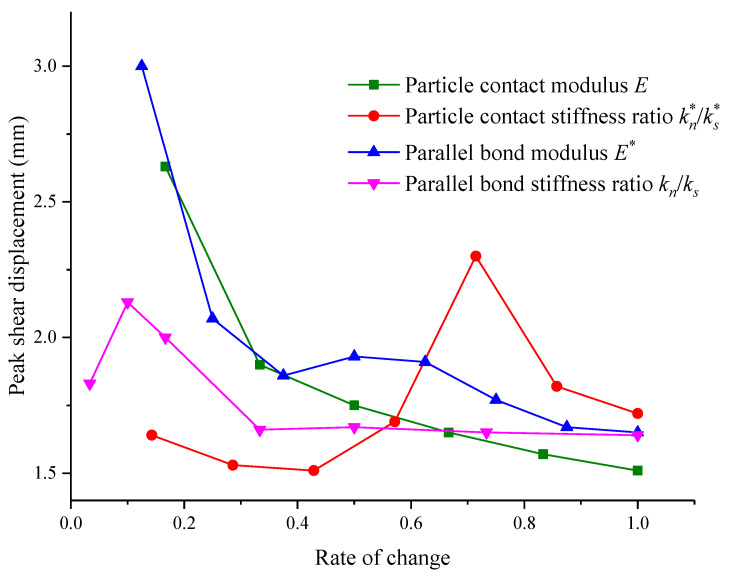
Relationships between micro-parameters and peak shear displacement.

Based on previous analysis, it is known that the contact modulus E of particles, contact stiffness ratio $k_{n}{}^{*}/k_{s}{}^{*}$ of particles, parallel bonding modulus $E^{*}$ and parallel bonding stiffness ratio $k_{n}{}^{*}/k_{s}{}^{*}$ all have a certain influence on the shear stress–shear strain pattern. The main reflection is on the peak shear strength and peak displacement. In order to intuitively reflect the relationships among several micro-parameters, peak shear strength and peak displacement of the structural plane, the corresponding influences on the shear strength and elastic modulus of different micro-parameters are summarized in Figure 11 and Figure 12.

As shown in Figure 11, the influences on shear strength of the contact modulus of particles, contact stiffness ratio and parallel bond modulus are small, but differences between these three factors exist. In general, the contact modulus and parallel bonding modulus can increase the shear strength. As the contact modulus increase, the corresponding shear resistance strength increases as well. When the change rate of contact modulus is 0.8, the shear resistance would be reduced a little. However, in general, the increment of contact modulus has a certain contribution to the incrementation of shear resistance strength. The increments in parallel bond modulus also contribute to the shear resistance strength, but when the change rate of the parallel bond modulus is more than 0.7, the shear resistance strength is lowered. As the contact stiffness increase, the shear resistance strength tends to be reduced again. Among the four micro-parameters, the parallel bond stiffness has the maximum influence on shear strength. As the change rate of parallel bonding stiffness increases, the shear strength is reduced. Additionally, the decrease rate of shear strength is the largest before the change rate of the bond stiffness ratio is less than 0.3. When it is higher than 0.3, the decrease rate of the shear resistance strength is reduced, and the curve slows down gradually.

As shown in Figure 12, the contact modulus and parallel bond modulus have the main influence on the shear stress peak. During the gradual increase in contact modulus, the peak displacement of shear strength is reduced to 1.5 from 2.6 mm. The influences on peak shear displacement had by increasing the parallel bonding modulus and contact modulus have similar tendencies. When the parallel bond modulus is increased gradually, the corresponding peak shear displacement is reduced to about 1.7 mm from 3.0 mm. During the process of parallel bond modulus incrementation, the curves of peak displacement–micro-parameters show small fluctuations. When the change rate of the parallel bond modulus is 0.5 or 0.6, the peak shear displacement is increased a little bit. When the change rate of the parallel bonding modulus is higher than 0.6, the peak shear displacement keeps reducing. The influence on shear strength of the parallel bonding stiffness ratio is mainly reflected in that when the change rate of the parallel bond stiffness ratio in the initial stage is less than 0.1, the peak shear displacement is increased. However, when change rate of the parallel bond stiffness ratio is higher than 0.3, the peak shear displacement begins to decrease, and later it remains stable. The influence on peak shear displacement had by contact stiffness is mainly = not obvious. In the beginning, as the contact stiffness ratio increases, the sheer strength is reduced tightly. Later, as the contact stiffness ratio increases, the peak shear displacement is increased as well. Finally, it appears to decrease as the contact stiffness ratio increases.

## 5. Quantitative Analysis of Micro-Parameters Based on Machine Learning

In order to further quantify the relationship between micro-parameters and macro-shear parameters, were used XGBoost and two other algorithms, PSO-XGBoost and WOA-XGboost, optimized by particle swarm optimization (PSO) and whale optimization algorithm (WOA), to process the data in Figure 11 and Figure 12. Then, with the coefficient of determination (R^2^) as the criterion, the algorithm with the best fitting effect was selected to calculate the weight share of each parameter and analyze the degrees of influence of micro-parameters to provide a theoretical reference for related studies.

### 5.1. Extreme Gradient Boosting Decision Tree (XGBoost)

XGBoost is an integrated classification algorithm and is called the extreme gradient boosting decision tree [40]. It is a distributed and efficient gradient boosting algorithm based on a decision tree (CART), and its basic idea is to combine several low-precision, weak classifiers into one high-precision classifier. Compared with similar boosting tree algorithms, XGBoost adds regular terms to the loss function and does second-order Taylor expansion on the loss function, which improves the computational accuracy and effectively avoids the overfitting of the algorithm. Moreover, due to the adoption of feature column sampling, the fitting effect is further improved and the computational effort of the algorithm is reduced.

The basic principle of the XGBoost algorithm is as follows [41]. For a given dataset $D=\left\{\left(X_{i},\;y_{i}\right)\right\}$, a model function is defined based on a classification decision tree (CART) as the base classifier:

(1) $${\hat{y}}_{i}=\phi (X_{i})=\sum_{k=1}^{K}f_{k}(X_{i})\;\left(f_{k}\in F\right)$$

Among them:

(2) $$F=\left\{f(X)=\omega_{q(X)}\right\}\left(q:{\mathbb{R}}^{m}\to T,\omega \in {\mathbb{R}}^{T}\right)$$

where K is the number of decision trees; F is the decision tree space; q(X) is the mapping of the sample X to the leaf nodes of the tree, whose corresponding leaf node fraction is $\omega_{q(X)}$; ${\mathbb{R}}^{m}$ is the m-dimensional real vector; T is the number of nodes of the corresponding tree; ${\mathbb{R}}^{T}$ is the T-dimensional real vector.

Define the objective function for model optimization, which consists of the loss objective function and the canonical term:

(3) $$\{\begin{array}{l} \mathrm{ojb}=\sum_{i=1}^{n}l(y_{i},{\hat{y}}_{i})+\sum_{i=1}^{n}Q(f_{k})\\ \Omega (f_{k})=\alpha T+\frac{1}{2}\lambda \sum_{j=1}^{T}\omega_{j}^{2} \end{array}$$

where ojb is the loss objective function; $l(y_{i},{\hat{y}}_{i})$ is the training error of sample $x_{i}$; ${\hat{y}}_{i}$ and $y_{i}$ are the predicted and actual ranks of sample $x_{i}$, respectively; $\Omega \left(f_{k}\right)$ is the canonical term of the kth classification regression tree; $\omega_{j}^{}$ is the weight of the jth leaf node; α and λ are the penalty coefficients.

The XGBoost algorithm randomly selects the training and test sets proportionally during the training process. When the process reaches the t-th round, the model’s objective function is as follows:

(4) $${\mathrm{ojb}}^{\left(t\right)}=\sum_{i=1}^{n}l[y_{i},{\hat{y}}_{i}^{t-1}+f_{t}(x_{i})]+\sum_{i=1}^{K}\Omega (f_{k})+C$$

where $f_{t}\left(x_{i}\right)$ is the t-th categorical regression tree added, and C is the complexity of the first t−1 trees.

An approximate expansion of Equation (4) with the second-order Taylor formula yields the approximate expression of the objective function as:

(5) $${\mathrm{ojb}}^{\left(t\right)}≃\sum_{i=1}^{n}l[y_{i},{\hat{y}}_{i}^{t-1}+g_{i}f_{i}(x_{i})+\frac{1}{2}h_{i}f_{i}^{2}(x_{i})]+\sum_{i=1}^{K}Q(f_{k})+C$$

where $g_{i}$ is the first order derivative of $l(y_{i},{\hat{y}}_{i}^{t-1})$ with respect to ${\hat{y}}_{i}^{t-1}$ and $h_{i}$ is the second order derivative of $l(y_{i},{\hat{y}}_{i}^{t-1})$ with respect to ${\hat{y}}_{i}^{t-1}$.

After simplification, the final objective function can be obtained as:

(6) $${\mathrm{ojb}}^{\left(t\right)}≃\sum_{i=1}^{T}[(\sum_{i\in I_{j}}g_{i})\omega_{j}+\frac{1}{2}(\sum_{i\in I_{j}}h_{i}+\lambda )\omega_{j}^{2}]+\alpha T$$

It solves the partial derivatives of the objective function $ojb^{\left(t\right)}$ with respect to $\omega_{j}^{}$ and makes the partial derivatives equal to 0. Then, the optimal weights that minimize the objective function are obtained as follows:

(7) $$\omega_{j}^{*}=\frac{\sum_{i\in I_{j}}g_{i}}{\sum_{i\in I_{j}}h_{i}+\lambda }$$

By substituting Equation (7) into Equation (6), the minimum value of the objective function is obtained as follows:

(8) $${\mathrm{ojb}}^{\left(i\right)}=-\frac{1}{2}\frac{{\left(\sum_{i\in I_{j}}g_{i}\right)}^{2}}{\sum_{i\in I_{j}}h_{i}+\lambda }+\alpha T$$

This determines the optimal structure of the t-th classification tree that minimizes the wood-variable function.

The XGBoost algorithm uses a random subspace method to select the optimal splitting point. At each splitting of a node, the feature values are randomly selected proportionally for different feature variables, and then each randomly selected feature value is traversed to select the splitting point that maximizes the gain function, which effectively improves the generalization ability of the model and avoids overfitting. In selecting the splitting point of the subtree, the gain function is defined as:

(9) $$\mathrm{Gain}=\frac{1}{2}[\frac{{\left(\sum_{i\in I_{L}}g_{i}\right)}^{2}}{\sum_{i\in I_{L}}h_{i}+\lambda }+\frac{{\left(\sum_{i\in I_{R}}g_{i}\right)}^{2}}{\sum_{i\in I_{R}}h_{i}+\lambda }-\frac{{\left(\sum_{i\in I_{j}}g_{i}\right)}^{2}}{\sum_{i\in I_{j}}h_{i}+\lambda }]-\alpha$$

where $\sum_{i\in I_{L}}g_{i}$ and $\sum_{i\in I_{L}}h$ are the gradient values of the left subtree of the splitting point; $I_{L}$ is the total set of splitting points of the left subtree; $\sum_{i\in I_{R}}g_{i}$ and $\sum_{i\in I_{R}}h_{i}+\lambda$ are the gradient values of the right subtree of the splitting point; $I_{R}$ is the total set of splitting points of the right subtree. The above calculations are continuously performed to determine the optimal structure of the new tree and the optimal splitting nodes, and the model’s prediction accuracy is improved by integrating the new tree.

### 5.2. Particle Swarm Optimization (PSO) and Whale Optimization Algorithm (WOA)

Particle swarm optimization (PSO) is an evolutionary computing algorithm derived from the study of bird predation behavior, which is inspired by the bird foraging process, and it has the characteristics of heuristics and random search of evolutionary algorithms [42]. In the PSO algorithm, birds work as particles, and the whole flock forms a particle swarm. As in other evolutionary algorithms, there are “groups” and “individuals” in the PSO algorithm. In the search process, each particle can be considered as an individual in the n-dimensional search space. The particles in the flight velocity can be dynamically adjusted according to the history and the optimal position of the historical population. The particles in the population have only two properties, i.e., speed (which denotes the moving speed) and position (denotes the direction of movement). The update equation for the velocity and position of each particle can be defined as follows:

(10) $$V^{\prime }=wV+c_{1}r_{1}\left(Pbest-M\right)+c_{2}r_{2}\left(Gbest-M\right)$$

(11) $$M^{\prime }=V^{\prime }+M$$

where Pbest and Gbest are the historical best position of a single particle and the historical best position of a particle swarm, respectively; the parameters $c_{1}$ and $c_{2}$ are called learning factors; $r_{1}$ and $r_{2}$ are the values of two random probability distributions in [0,1]; w is the inertia weight; M and V denote the current position and velocity of the particle, respectively; and the updated position and velocity of the particle are denoted by $M^{\prime }$ and $V^{\prime }$, respectively.

The optimal solution of each particle search is called the individual extreme value, and the individual extreme value in the particle swarm is taken as the current global optimal solution. Then, it iterates continuously to update the speed and position, and finally gets the optimal solution that satisfies the termination condition. In this process, each particle collaborates with the others to better adapt to the environment and achieve the optimal search for complex solutions in complex spaces.

The whale optimization algorithm (WOA) is a population-based algorithm inspired by humpback whales hunting for prey [43]. It uses a unique technique based on a special bubble net and the behavior of feeding whales. To capture prey, the whales have three action steps: (1) encircling the prey; (2) exploitation (foam net attack); (3) exploration (find prey). The details of the WOA can be found in the following study [44].

### 5.3. Model Validation and Analysis

**Figure 13:**
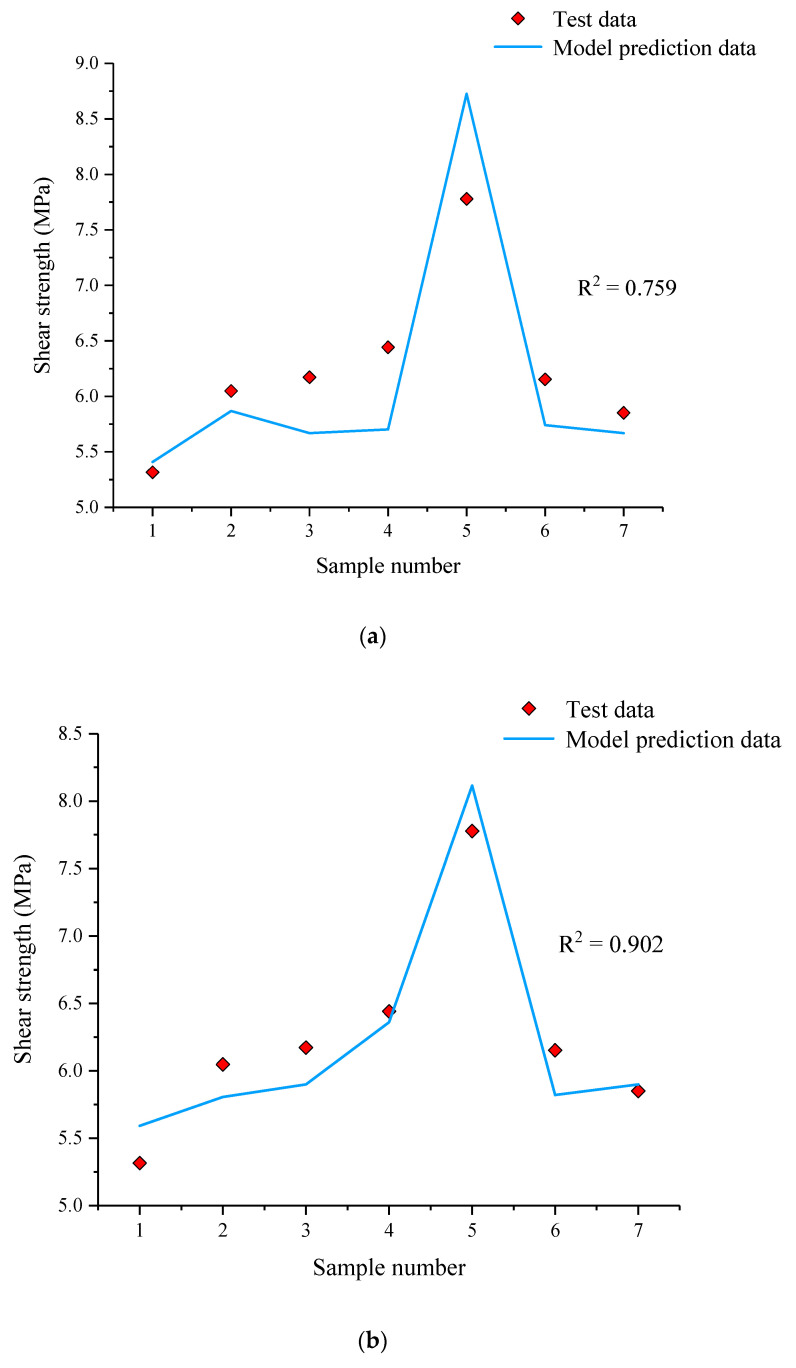

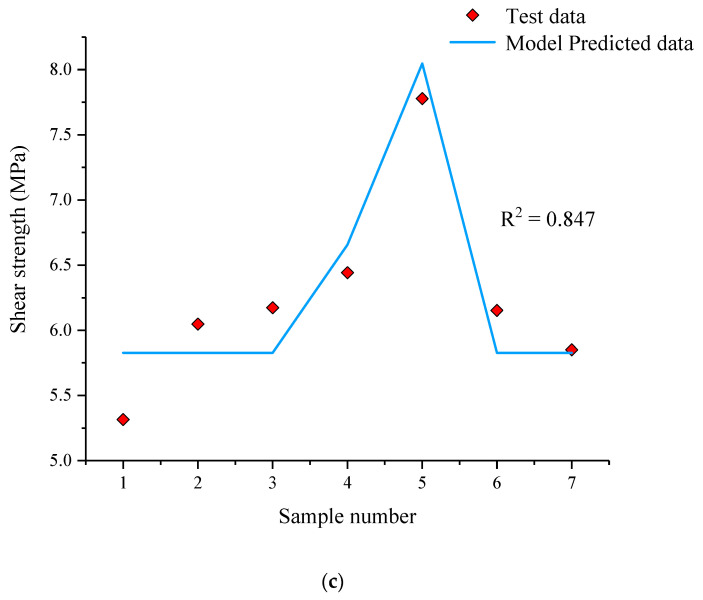
The comparison between the test data and predicted data being used by different models: (**a**) XGBoost, (**b**) WOA-XGBoost, (**c**) PSO-XGBoost.

To explore a better way to study the relationship between microscopic parameters and shear strength, we combined two optimization algorithms (PSO and WOA) with XGBoost to construct two hybrid intelligent models, PSO-XGBoost and WOA-XGBoost. Together with the original XGBoost model, there were three intelligent models in total. Then, 75% of the data in Figure 11 and Figure 12 were used as the training set to train the intelligent prediction model. Finally, the remaining 25% of the data were compared with the predicted data of the model to test the prediction accuracy of the model, as shown in Figure 13.

**Figure 14:**
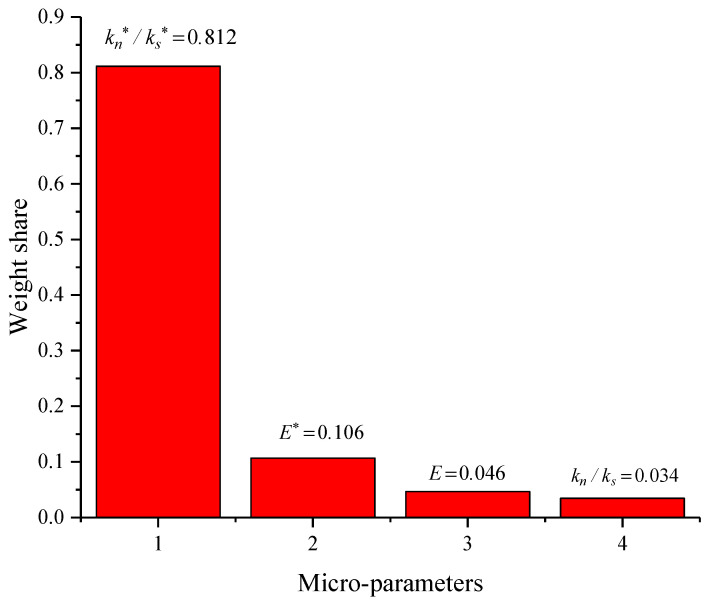
Weight shares of different microscopic parameters for shear strength.

In Figure 13, it can be realized that XGBoost had the lowest prediction accuracy with an R^2^ of 0.759, and WOA-XGBoost has the highest prediction accuracy and the best agreement between the predicted data and test data with an R^2^ of 0.902. PSO-XGBoost had a prediction accuracy between those of XGBoost and WOA-XGBoost with an R^2^ of 0.847. WOA-XGBoost achieved a very good fit between the predicted data and the test data for shear strength. Therefore, the WOA-XGBoost model was selected as the prediction model in this study, and the weight share of each micro-parameter was calculated based on the weight calculation module of the algorithm, and the results are shown in Figure 14.

As can be seen in the figure, the weight share of $k_{n}{}^{*}/k_{s}{}^{*}$ is the highest, reaching 0.812, followed by $E^{*}$ at 0.106, and the weight shares of E and $k_{n}/k_{s}$ are the lowest, adding up to less than 0.1. This shows that $k_{n}{}^{*}/k_{s}{}^{*}$ is the factor that has the greatest influence on shear strength; $E^{*}$ has a small influence; and E and $k_{n}/k_{s}$ have almost no influence.

## 6. Conclusions

In this study, to investigate the crack evolution law and the effects of microscopic parameters on the serrated structural plane, a series of numerical simulations of direct shear tests based on PFC2D were carried out. The main conclusions are as follows:

(1) The relationships among the number of microcracks, the normal stress and the shear stress during the shearing process of the serrated structural plane were analyzed. The results show that the greater the normal stress, the higher the number of microcracks. Before the shear stress reached its peak, the number of cracks increased slowly; after the shear stress reached its peak, the degrees of crack sprouting and expansion increased, the number of cracks kept increasing and the damage accumulated. Eventually, when the shear stress reached the residual shear strength, the number of microcracks tended to stabilize.
(2) During the shearing process, the contact force distribution on the serrated surface has similarity with the evolutionary law of microcracks. In the initial stage of shearing, the contact force chain is mainly concentrated at the root of the sawtooth. As the shear progresses, the contact force chain becomes more and more concentrated, and the force on the root of the sawtooth becomes larger and larger. When the failure finally occurred, a large number of cracks appeared at the root of the sawtooth.
(3) The four meso-parameters (particle contact modulus E, particle contact stiffness ratio $k_{n}{}^{*}/k_{s}{}^{*}$, parallel bond modulus $E^{*}$, and parallel bond stiffness ratio $k_{n}/k_{s}$) have certain influences on the shear stress–shear displacement curve, shear strength and peak shear displacement. The particle contact stiffness ratio $k_{n}{}^{*}/k_{s}{}^{*}$ and parallel bond stiffness ratio $k_{n}/k_{s}$ are negatively correlated with the shear strength; and the particle contact modulus E and parallel bond modulus $E^{*}$ are positively correlated with the shear strength. The peak shear displacement gradually decreases as the particle contact modulus E and parallel bond modulus $E^{*}$ increase. The parallel bond stiffness ratio $k_{n}/k_{s}$ was negatively correlated with the peak shear displacement to some extent. The effect of parallel bond stiffness ratio $k_{n}/k_{s}$ on the peak shear displacement was not significant.
(4) To further quantitatively study the relationships between micro-parameters and shear strength, XGBoost, WOA-XGBoost, and PSO-XGBoost algorithms were introduced to construct the quantitative prediction model. It was found that WOA-XGBoost had the highest prediction accuracy and the best agreement between the predicted data and test data with an R^2^ of 0.902. Using this model to calculate the weight shares of micro-parameters, we realized the weight share of $k_{n}{}^{*}/k_{s}{}^{*}$ should be the highest, 0.812, followed by $E^{*}$ at 0.106, and the weight shares of E and $k_{n}/k_{s}$ were the lowest, adding up to less than 0.1. This shows that $k_{n}{}^{*}/k_{s}{}^{*}$ is the factor that has the greatest influence on shear strength; $E^{*}$ has a small influence; E and $k_{n}/k_{s}$ have almost no influence.

Finally, it should be noted that this research only studied the crack evolution law and micro-parameter analysis of serrated planes by the discrete element method, without mechanical analysis based on other numerical methods, such as the finite element method, and also lacked in-depth verification at the experimental level and mechanical theoretical analysis. Moreover, more in-depth studies, such as the study of mechanical properties under the coupling of various factors, such as water, temperature and pH, are not enough. These are the directions for further research in the future.

## Figures and Tables

**Table 1:** Micro-mechanical parameters of the structural plane pattern.

Min Particle Diameter (mm)	Particle Radius Ratio	Contact Modulus (GPa)	Contact Stiffness Ratio	Bond Modulus (GPa)	Bond Stiffness Ratio	Parallel Bond Normal Strength (MPa)	Parallel Bond Cohesion (MPa)	Internal Friction Angle of Parallel Bonding
0.4	1.6	4	2.1	0.08	2.2	7.2	23.56	53.3

**Table 2:** Shear mechanical parameters under test and simulation conditions.

	Test Condition	Simulation Condition
Cohesion (MPa)	2.466	1.713
Internal friction angle (°)	55.55	64.21

## Data Availability

The data used to support the findings of this study are available from the corresponding author upon request.
